# Supplementary material for: GIS-based land suitability evaluation and multi-criteria decision analysis for sustainable enset (Ensete ventricosum (Welw.) Cheesman) cultivation in Hadiya Zone, Central Ethiopia
Source: PLoS One. 2026 Mar 9;21(3):e0344127. doi: 10.1371/journal.pone.0344127 (PMC12970975; doi:10.1371/journal.pone.0344127)
Supplement: Attachment — Submitted filename: Responses.pdf [file pone.0344127.s004.pdf]

**Dear Editors;** We sincerely thank you for your careful evaluation of our revised manuscript and for highlighting the missing attribution details in the captions for Figures 1, 12, and 13 in accordance with PLOS copyright policy.

In response to your comment, we have now fully updated the figure captions to include the required source attributions and licensing information, in compliance with the relevant data licenses. Specifically:

- **Figure 1** now includes attribution to the Humanitarian Data Exchange (HDX) dataset *"Ethiopia Administrative Boundaries (COD-ABETH)"*, with explicit reference to the Creative Commons Attribution 4.0 International (CC BY 4.0) license and appropriate authorship attribution.

| **Editorial Comment** | **Response** | **Action Taken** | **Location in Manuscript** |
|---|---|---|---|
| The captions for Figures 1, 12, and 13 do not yet include the required attribution associated with maps made using data from the sources you mentioned. Please refer to the license associated with the data used to make these maps and update your attributions in the Figure captions accordingly | We thank the editors for this important observation. The figure captions have been fully revised to include complete source attribution and licensing information in accordance with PLOS ONE copyright policy and the respective data licenses | **Figure 1:** Attribution added for the Humanitarian Data Exchange (HDX) dataset *"Ethiopia Administrative Boundaries (COD-ABETH)"* with explicit reference to the Creative Commons Attribution 4.0 International (CC BY 4.0) license and "Adapted by the authors." **Figure 12:** Attribution added for the World Bank Climate Knowledge Portal, acknowledging the use of open-access climate datasets derived from publicly available global and national sources. **Figure 13:** Attribution added for the World Bank Climate Knowledge Portal, acknowledging the use of open-access climate datasets derived from publicly available global and national sources. | Figure captions for **Figure 1**, **Figure 12**, and **Figure 13** |

- **Figures 12 and 13** now include proper attribution to the World Bank Climate Knowledge Portal, with acknowledgment of the use of open-access climate datasets derived from publicly available global and national sources, in line with the applicable open data use policies.

All captions have been revised to ensure full compliance with PLOS ONE copyright and attribution requirements.

We appreciate your guidance in this matter and thank you for the opportunity to correct these details to meet the journal's publication standards.

Sincerely,
**Alemu Ersino Ersado**
