# Supplementary material for: GIS-based land suitability evaluation and multi-criteria decision analysis for sustainable enset (Ensete ventricosum (Welw.) Cheesman) cultivation in Hadiya Zone, Central Ethiopia
Source: PLoS One. 2026 Mar 9;21(3):e0344127. doi: 10.1371/journal.pone.0344127 (PMC12970975; doi:10.1371/journal.pone.0344127)
Supplement: Attachment — Submitted filename: Response to Reviewer.docx [file pone.0344127.s002.docx]

**Dear Editor and Reviewers:**

I wish to express my genuine gratitude for your invaluable remarks, insightful comments, and helpful recommendations regarding our work submitted to PLOS ONE. The comments from the editor and two reviewers has been invaluable, providing a wonderful opportunity to enhance the clarity, quality, and scientific rigor of our work. We have carefully amended the manuscript in alignment with all comments and recommendations. All modifications have been integrated into the pertinent portions of the article, and comprehensive, itemized responses to each comment are included in this document. We appreciate your time and effort in reviewing our work and believe that the new version has been considerably enhanced due to your insightful feedback. I greatly appreciate your consideration.

Respectfully, Alemu Ersino Ersado and (On behalf of co-author)

**ACKNOWLEDG EMENTS**

The authors gratefully acknowledge the Department of Geography, Andhra University, for their invaluable support in facilitating this research on enset in Ethiopia. We also extend our sincere appreciation to Wachemo University for their cooperation and continuous encouragement throughout the study. Finally, our heartfelt thanks go to all respondents and key informants in the Hadiya Zone for generously sharing their time and insights.

**Declaration of Competing Interest**

The authors declare that they have no known competing financial interests or personal relationships that could have appeared to influence the work reported in this paper

**Funding declaration**:

This research received no specific grant from any funding agency in the public, commercial, or not-for-profit sectors. No funding was received

**Ethics Declaration**

Ethical approval was not required for this study as it did not involve human participants or animals.

**Data Availability Statement**

All data generated or analyzed during this study are included in this published article. Additional data can be provided by the corresponding author upon reasonable request.

Q3. **Regarding the permits you obtained for the work**

For this research, official authorization and letters of collaboration were secured from Andhra University, India, where I am undertaking my PhD, and from Wachemo University, Ethiopia, my home university. These letters enabled official partnership and granted permission to perform fieldwork in the study areas. In light of these institutional supports, supplementary oral approvals were obtained from the Hadiya Zone Agricultural Office and the corresponding District (Woreda) Agricultural and Natural Resource Management Offices in Central Ethiopia. The authorities granted access to the field location and supplied the requisite data and expert collaboration for the study operations.

**Q4. All PLOS journals now require all data underlying the findings described in their manuscript to be freely available to other researchers**

I am grateful for your message and for the opportunity to provide some light on the matter. I agree to the fact that the published article contains all of the data that was gathered and examined throughout the process of this research. The author of this study gathered all of the measurements, satellite images, and field observations that were used to compile the datasets that were utilized in this research. All of the relevant information has been thoroughly evaluated, interpreted, and presented in the sections of the text that are devoted to the results and the discussion. As a result, there are no more statistics or data that have not been submitted that are beyond what is provided in this article. Nevertheless, due to data usage agreements and access restrictions, certain supporting materials, such as satellite images and source papers received from institutional or third-party databases, are not accessible to the general public and cannot be shared with them.

Q5….

Q6. Please ensure that you refer to Figure 1,2,4,5,6,9,11,12,13 and 14 in your text as, if accepted, production will need this reference to link the reader to the figure. Revised accordingly

Q7. Please include a caption for figure 14. Included a caption

Q8. You may seek permission from the original copyright holder of Figure(s) [#] to publish the content specifically under the CC BY 4.0 license.

All the information included in this manuscript has been clearly described in the Methods section, with details provided on data sources and types. The authors created all of the maps and pictures included in the publication. Any copyright issues are entirely the authors' responsibility. Additional permits from any external authority were not required because the satellite images and other datasets used in this work were sourced from open-source and freely available platforms.

Q9. We note you have included a table to which you do not refer in the text of your manuscript. Please ensure that you refer to Table 1, 3,4,5,6,10,11,12 and 14 in your text; if accepted, production will need this reference to link the reader to the Table. **As required tables included and refered under the manuscript.**

**Comments of Reviewers**

**Reviewer #1**

1. **What was the selection of criteria or factors based on? Please explain.**

I appreciate your significant inquiry. There is no universally accepted method for analyzing the suitability of land for enset cultivation that works in every situation,(Mugiyo et al., 2021). Rather, the approach is modified in accordance with the unique environmental circumstances, contextual elements, and resource accessibility of the specific study region.

As a result, determining the appropriateness levels for each criterion depends on the context and incorporates information from multiple sources. These include published research findings from research institutions, pertinent scientific literature about enset farming, the author's own field-based experience, national agricultural manuals and guidelines, and opinions from subject matter experts. This multi-source approach guarantees that the suitability assessment incorporates both locally relevant knowledge and information that has been verified by science. The criteria selection was grounded in a thorough examination of the current scientific literature regarding enset (Ensete ventricosum) ecology and agronomy, as well as professional knowledge and personal experiences. These parameters have been repeatedly recognized as the principal determinants of enset development, production, and sustainability. These factors climatic, soil, topography, and land use/land cover parameters are acknowledged as the primary variables affecting the growth, productivity, and distribution of enset, FAO. (1976), FAO. (2007), Woldetsadik, et al., (2017), Bantider, A., & Hurni, H. (2007).

Generally, the major suitability factors considered: **Climate** (Temperature, Rainfall): Directly influences enset's physiological functions, frost resilience, and hydration needs. **Soil characteristics** (depth, pH, organic carbon, texture, type) are essential for root development, nutrient availability, water retention, and general plant health, as evidenced by several research on enset. **Topography** (slope, elevation) serves as an indicator of temperature (through lapse rate) and drainage, impacting soil erosion risk and agricultural management practices. **Land Use/Land Cover (LULC**): Crucial for recognizing existing land and comprehending human-induced stresses, guaranteeing that the suitability model accurately represents real-world limitations. This multi-criteria approach implies that our model encompasses the comprehensive environmental prerequisites for effective enset cultivation.

2. **In the discussion section, it is better to make comparisons with the researches of others	and the research done**

We greatly appreciate this useful suggestion. In accordance with your request, we have amended the discussion section by incorporating comparisons with data from many related studies to enhance the interpretation of results and offer a broader scientific context. We have specifically included relevant findings publications, such as (Mugiyo et al., 2021), (Amiri & Nateghi, 2023), (Amiri & Nateghi, 2023) and (Yohannes &Soromessa, 2018), (Ayanu et al., 2015), ,(Seyedmohammadi & Naser, 2022), (Gelalacha et al., 2024), (Jarasiunas, 2016), (Kabir et al., 2025).

3. **Please provide valid references for the relationships provided;**

We appreciate your constructive feedback. We have incorporated relevant and current references that support the linkages outlined in the article, as recommended. The updated edition now incorporates pertinent citations from recent and relevant studies to enhance the credibility of the arguments articulated.

4. **Why have properties such as lime, gypsum, and exchangeable sodium not been examined in examining soil properties?**

Thank you for this significant observation. This study selected soil parameters based on their direct relation to enset cultivation requirements and the availability of credible data in the study area. Soil texture, depth, pH, organic carbon content, and type were identified as the primary elements influencing enset growth, productivity, and site suitability. These criteria are well acknowledged in prior land suitability and crop growth research as critical factors influencing soil fertility and root development. The exclusion of properties such as lime, gypsum, and exchangeable sodium was mostly due to:

a. Their influence is comparatively negligible in the context of enset cultivation relative to the chosen parameters.
b. Data limitations consistent and accurate field or laboratory measurements for certain parameters were unavailable across all sampling locations.
c. The study aimed to identify the most ecologically and agronomically pertinent characteristics for regional-scale land suitability evaluations, rather than doing a comprehensive assessment of soil chemical amendments.

We recognize the significance of these features in affecting soil chemical equilibrium and advise that future research include them for a more thorough evaluation.

5. **The advantages and disadvantages of the research done should be said.**

This study possesses several advantages, notably the integration of field-based soil data with GIS-based land suitability analysis, thereby expanding the reliability and spatial precision of the results. Additionally, it yields significant insights into the ecological viability of enset cultivation across diverse biophysical conditions in the Hadiya Zone. However, a few limitations warrant attention. The analysis excluded specific soil chemical properties such as lime, gypsum, and exchangeable sodium due to a lack of data, and it depended on climate data from a restricted temporal scope. Despite these limitations, the findings provide an established basis for sustainable agricultural planning and future comprehensive investigations.

6. **In the AHP method, did you use the opinions of relevant** **experts in the form of a questionnaire to determine the importance of the factors in the pairwise comparison matrix?**

In fact, we did. In the Analytical Hierarchy Process (AHP), expert opinions were systematically gathered through a structured questionnaire for determining the relative importance (weights) of the selected elements in the pairwise comparison matrix. The experts were deliberately chosen based on their professional expertise and academic qualifications in the fields of soil science, agronomy, land use planning, and geography. Each expert was requested to evaluate the criteria—namely climate, soil qualities, terrain, and land use/land cover—according to their perceived impact on the appropriateness for enset farming. Pairwise comparisons were performed utilizing the Saaty (1980) scale of relative importance, which ranges from 1 to 9. The consolidated expert evaluations were subsequently synthesized to calculate the normalized weights and consistency ratio (CR) to guarantee logical coherence and dependability of the comparisons (CR < 0.1). The CR value was computed using Expert Choice software. Since the obtained CR value (0.0342 or 3.4%) is well below the generally accepted threshold of 0.10 (10%), the pair-wise comparison matrix is considered consistent. Therefore, no further revision of the judgment matrix was required.

7. **Please use the papers (**[**https://doi.org/10.1007/s10661-022-10327-x ...............etc**](https://doi.org/10.1007/s10661-022-10327-x%20...............etc)

Yes, we used to improve the quality of the manuscript

8. **Please give the names of soils according to the WRB system.**

Thank you for your insightful remark. This study identified and classified soil types according to the World Reference Base for Soil Resources (WRB, FAO 2022) approach. The classification employed field observations, soil profile descriptions, and secondary data from national soil information sources. The principal soil units recognized in the research region comprise Eutric Nitisols, Vitric Andosols, Chromic Nitisols, Pellic Vertisols, Chromic Luvisols, Eutric Fluvisols, Eutric Cambisols, Chromic Cambisols, Dystric Nitisols, Lithic Leptosols, Calcisols/Xerosols, and Orthic Solonchaks, (WRB, 2022),(Mantel et al., 2023).

9. **What was the accuracy of the methods used? By which criteria are the methods evaluated?**

**Model Validation and Accuracy Evaluation**

This study utilized two validation procedures to ensure the validity and accuracy of the spatial analyses: Suitability Model Validation and Land Use/Land Cover (LULC) Classification Accuracy Assessment.

**1. Validation of Suitability Model**

GPS-based ground control points obtained from established enset production sites were superimposed on the suitability map to assess the model's predicted efficacy. A confusion matrix was created, and statistical metrics like overall accuracy and the Kappa coefficient were calculated to assess the relationship between the anticipated and actual appropriateness classes. These indices offered a quantitative evaluation of the model's precision in identifying appropriate regions for enset cultivation within the research area (Congalton & Green, 2019; Foody, 2002).

**2. Evaluation of LULC Classification Accuracy**

Forty-eight ground truth reference locations were utilized to confirm the LULC classification. The points were randomly chosen and validated by high-resolution imagery from Google Earth Pro and field observations performed in the Hadiya Zone. The points were allocated across primary land cover categories as follows: 18 from agricultural land, 14 from forest and shrubland, 8 from grassland, 4 from urban areas, 2 from barren land, and 2 from aquatic environments and wetlands. A confusion matrix was created to calculate overall accuracy, producer's accuracy, and user's accuracy for every land use category. The classification attained an overall accuracy of 94% and a substantial Kappa coefficient, indicating robust concordance between the categorized map and the reference data. The results validate the dependability of the final LULC map utilized for the analysis.

10. **Please check the grammar of the whole text with a native speaker and fix the errors.**

We have made every effort to correct all grammatical errors to the best of our ability

**Reviewer #2: General Overview**

Major Points for Revision and Clarification
**1. Title and Abstract**: Required revision has made.

**2. Methodology Section**: To eliminate uncertainty, the datasets displaying the best spatial resolution and most uniform coverage for the research region were chosen for final analysis. Climatic variables, including station-based temperature and rainfall data sourced from the National Meteorological Agency (NMA), were interpolated to a 1 km² grid resolution via the Inverse Distance Weighting (IDW) method. ERA5 data were included solely for verifying consistency and were not incorporated into the final simulation. Among the accessible DEM sources (SRTM 10 m and ASTER 30 m), ASTER GDEM v3 (30 m) was chosen for its broader local coverage and compatibility with additional datasets. Landsat 8 (2022) imagery was utilized for land use and land cover mapping due to its spectrum appropriateness and comparatively high spatial resolution (10 m). Soil and administrative data were aggregated from national and local sources and standardized into a 1 km² raster format for spatial analysis.

**-Criterion Weighting: “**The process for obtaining the pairwise comparisons (the expert judgments) is not described. How many experts were involved? What was their field of expertise?”

The significance of each appropriateness factor was assessed utilizing the Analytical Hierarchy Process (AHP) established by Saaty (1980). Expert judgment was utilized to produce pairwise comparisons among the selected criteria. Seven specialists engaged in the weighting process, comprising two agronomists, two soil scientists, one environmental management specialist, one GIS expert, and one agricultural officer from the local agricultural bureau. All participants demonstrated comprehensive expertise in enset-based agricultural systems and land suitability evaluation within the Hadiya Zone. Each of the experts independently assessed the significance of the criteria specifically climatic factors (temperature and precipitation), soil properties (soil depth, soil type, soil pH, and organic carbon), topographic variables (slope and elevation), and land use/land cover (LULC) utilizing Saaty’s nine-point scale (1–9).

The various pairwise comparison matrices were normalized and averaged to generate the final composite matrix. The eigenvector approach was employed to determine the relative weights for each criterion from this matrix. The Consistency Ratio (CR) was calculated to evaluate the internal consistency of judgments, yielding a CR value below 0.1, which signifies an appropriate degree of consistency. The calculated weights were subsequently utilized in the weighted overlay analysis to produce the final enset suitability map.

**-Suitability Classification Thresholds:** The manuscript would be significantly strengthened by including a table or a section: We genuinely value the reviewer's helpful comments and suggestions. However, not all elements of the extensive research could be included in the publication due to constraints of scope and space. Certain criticisms raised by the reviewer relate to sections included in the author's main PhD thesis, although they exceed the precise aims of this essay. This article offers a concise summary of the extensive criteria, data range specifications, and comprehensive suitability classification table for enset cultivation, which are thoroughly described in the thesis to ensure clarity and focus.

Criteria used, data range, and suitability class for enset

| **Criteria** | **H (S1)** | **(S2)** | **(S3)** | **(N1)** | **(N2)** |
| --- | --- | --- | --- | --- | --- |
| **Soil Depth** | >1m | 0.8–1m | 0.6–0.8m | 0.6 - 0.5m | <0.5m |
| **Soil Texture** | clay- loam | Loam | sandy clay loam | Sandy-loam |  |
| **Soil type** | Eutric Nitisols, Vitric Andosols &Chromic Nitisols | Pellic Vertisols, **Chromic Luvisols &**  Eutric Fluvisols | Eutric Cambisols,  Chrom0ic Cambisols & Dystic Nitisols | Lithic Leptosols& Calcisols/Xerosols | Orthic Solonchaks |
| **pH Range** | 5.5–7.5 | 5.0–5.4 or 7.6–8.0 | 4.5–4.9 or 8.1–8.5 | <4.5 or >8.5 |  |
| **Soil OC** | >4% | 2–3% | 1-2% | < 1% | **-** |
| **LULC** | Crop land | Settlement | Forest land | Grassland/wetland/Bared | Baredland/water |
| **pH Range** | 5.5–7.5 | 5.0–5.4 or 7.6–8.0 | 4.5–4.9 or 8.1–8.5 | <4.5 or >8.5 |  |
| **Rainfall (mm)** | >1,350 | 1150-1350 | 950-1150 | 800-950 | <800 |
| **Temperature (°C)** | 15-17 | 17-20 or 12-15 | 20-23 | <12 or 23-26 | >26 |
| **Slope (%)** | <10 | 10-12 | 12-15 | 15-18 | >18% |
| **Elevation** | >2,100m | 2,100–1,900m | 1,900- 1700 | 1700-1600 | <1500 |

**3. Results and Discussion Section:** The results are presented well for each individual factor, but the discussion of the integrated overall result (Section 3.3) is relatively brief.

We developed the suitability classification for each parameter that was used in evaluating the potential of the study region for enset cultivation by conducting a systematic comparison between the spatial distribution of each environmental element and the established enset crop requirement standards. This comparison was utilized to determine whether or not the study area was suitable for enset cultivation. Temperature, rainfall, soil depth, soil texture, soil type, soil organic carbon (SOC), soil pH, elevation, slope, and land use/land cover (LULC) were some of the elements that were taken into consideration during this investigation.

For every parameter, the relevant values within the research region were overlaid with ecological thresholds that were specific to the enset. These criteria were obtained from academic literature and conversations with specialists. According to (FAO, 2076) land

evaluation framework, each factor was classified into one of five suitability classes based on these comparisons. These grades were as follows: Very Highly Suitable (S1), Highly Suitable (S2), Moderately Suitable (S3), Marginally Suitable (N1), and Permanently Not Suitable (N2). The detection of geographical variations in suitability for enset cultivation was made possible by this classification, which also served as a platform for further multi-criteria evaluation and weighted overlay analysis.

- **Where are the S1 areas concentrated? Do they correlate with specific districts?**

The regions designated as Very Highly Suitable (S1) for enset cultivation are primarily located in the middle and northern sections of the study area. These zones mostly pertain to the Duna, Misha, Lemo, and portions of the Soro districts within the Hadiya Zone. The exceptional appropriateness in these districts is chiefly due to their advantageous agro-climatic conditions, which encompass moderate to high precipitation, ideal temperature ranges, deep and well-drained soils rich in organic matter, and gently sloping to moderately undulating terrain.

- **Discuss potential trade-offs. For example, an area might have highly suitable soil but a less suitable slope. How does the model handle this?**

In multi-criteria land suitability analysis, it is common for specific components to exhibit different suitability levels within the same region. A location may have deep, fertile soils (highly appropriate) yet be situated on a steep slope (less suitable), resulting in trade-offs in the overall suitability assessment. This study utilized the Analytical Hierarchy Process (AHP) combined with a weighted overlay method in GIS to solve these trade-offs. Each parameter (e.g., soil, slope, rainfall, temperature) was allocated a relative weight according to its significance for enset cultivation, as assessed by expert evaluation. The weighted overlay procedure combines all reclassification layers by multiplying each factor's normalized appropriateness score by its designated weight and aggregating the outcomes.

This technique enables the model to assign greater significance to influential elements (e.g., climate variables, soil type, and land use/land cover) in determining the final suitability score, while less important aspects (e.g., soil pH and slope, depending on weight) exert comparatively minor effects. Thus, even if one element is less optimal, regions with advantageous conditions in other significantly weighted criteria may still attain a high overall appropriateness categorization.

- **On Page 24 and 25 the text state the total area is 3,635.5 km², with 57.72% (2,098.79 km²) as S1.** The correction has made “2088.79 km^2^ (57.72%), 1339.81 km^2^ (36.89%), 19.0 km^2^ (0,159%) and 188.21 km^2^ **(**5.231%)”
- The figures referenced in the text (Fig. 1, 2, 3, etc.) are not included in the provided PDF in a viewable format. The authors must ensure all figures are of high quality, with legible legends, scales, and north arrows.

**Everything is clearly feasible**

Fig 1. Map of the study area

Figure 2. Flow diagram of the method used for suitability analysis (chart)

Figure 3. Current reclassified land use type in the study area (pie chart)

- **Minor Corrections and Suggestions**

I would like to express my sincere appreciation to the reviewer for their valuable comments, minor corrections, and constructive suggestions. All the feedback provided by the reviewer has been carefully considered and appropriately incorporated into the revised manuscript and the accompanying response report.
