# Supplementary material for: GIS-based land suitability evaluation and multi-criteria decision analysis for sustainable enset (Ensete ventricosum (Welw.) Cheesman) cultivation in Hadiya Zone, Central Ethiopia
Source: PLoS One. 2026 Mar 9;21(3):e0344127. doi: 10.1371/journal.pone.0344127 (PMC12970975; doi:10.1371/journal.pone.0344127)
Supplement: Attachment — Submitted filename: Response to the revieweers.docx [file pone.0344127.s003.docx]

**Response to the Editors and Reviewers**

I would like to sincerely thank the Editors and Reviewers of PLOS ONE for their valuable time, constructive comments, and insightful suggestions. I have learned greatly from your expertise throughout the review process. I truly appreciate every comment provided, and all points raised by the Editors and Reviewers have been carefully addressed. The detailed responses are provided below, point by point.

1. **Heading styles**: All heading and subheading styles throughout the manuscript have been carefully reviewed and revised to strictly conform to the PLOS ONE journal formatting and style guidelines.
2. **Financial disclosure**: There are no changes to the financial disclosure statement. The originally submitted disclosure remains accurate and complete, and no conflicts of interest are declared.
3. **Titles and subtitles**: All section titles and subtitles have been revised for clarity, consistency, and alignment with the reviewers’ comments, ensuring improved readability and coherence.
4. **Reference list**: The entire reference list has been thoroughly reviewed to ensure completeness, correctness, and consistency with PLOS ONE citation guidelines. All references have been verified to the best of my knowledge.
5. **Figures (general formatting)**: All figures have been carefully reviewed, adjusted, and properly aligned to enhance visual clarity and consistency across the manuscript.
6. **Methodology figure**: The methodology figure has been improved to enhance clarity, logical flow, and interpretability, as suggested by the reviewers.
7. **Uniform text style in figures**: All figures now use a uniform text style (font type and size) and have been revised in accordance with the reviewers’ specific comments.
8. **Figure legends and readability**: All figure legends have been revised to ensure they are clear, complete, and easily readable, with sufficient explanation for standalone interpretation.
9. **Figure headings**: Figure headings have been removed and appropriately incorporated into the figure legends, following PLOS ONE guidelines.
10. **Equation numbering**: All equations have been reviewed, and consistent numbering has been maintained throughout the manuscript.
11. **Technical soundness and data support**: In response to the question “Is the manuscript technically sound, and do the data support the conclusions?”, one reviewer responded “No” while the other responded “Yes.” I agree with the positive assessment. The manuscript presents technically sound scientific research, and the data adequately support the conclusions. The experiments were conducted rigorously, with appropriate controls, replication, and sample sizes.
12. **Statistical analysis**: Regarding the question “Has the statistical analysis been performed appropriately and rigorously?”, one reviewer responded “N/A” and the other “Yes.” The statistical analyses in this study were conducted appropriately and rigorously, using suitable methods aligned with the study objectives and data structure.
13. **Figure quality and NAAS tool**: All figures were further adjusted using the free NAAS figure preparation tool, as recommended by the reviewers. This tool was used to assess and improve figure quality and ensure compliance with PLOS ONE technical requirements by comparing each figure against the journal’s specifications.
14. **Recommended articles**: Among the recommended articles, one was incorporated into the manuscript. However, access to the full PDF of recommended articles were limited, which constrained further detailed review and incorporation.
